# YBX1/CD36 positive feedback loop: novel molecular target for MASLD treatment

**DOI:** 10.7150/ijbs.121866

**Published:** 2025-08-11

**Authors:** Yuxuan Jiang, Cristina Llorente

Metabolic dysfunction-associated steatosis liver disease (MASLD), affecting over one-third of adults worldwide, poses a growing global health challenge 1. Although numerous molecules, including thyroid hormone receptors β (THRβ) and fibroblast growth factor 21 (FGF21), have been tested as potential targets for treating MASLD, few have demonstrated sufficient efficacy or acceptable side-effect profiles 2. Therefore, identifying novel molecular targets for MASLD treatment is of critical importance.

Y-box binding protein 1 (YBX1) is a multifunctional DNA- and RNA-binding protein that plays an essential role in gene expression regulation 3. Due to its critical role in apoptosis regulation, YBX1 has been associated with the development of various cancers. For example, Lee et al. demonstrated that YBX-1 protects breast cancer cells from apoptosis by participating in the STAT3 pro-survival signaling pathway 4. In addition, the roles of YBX1 in metabolism are also progressively being elucidated. Sakaguchi et al. showed that YBX1 plays a key role in preserving insulin sensitivity in adipocytes 5. These findings raise the question of whether YBX1 may also contribute to lipid accumulation in the liver during MASLD.

Supporting this hypothesis, a recent study by Zhang et al. has demonstrated the role of YBX1 in MASLD, by functioning as a transcription factor in hepatocytes, upregulating CD36 expression and establishing a positive feedback loop that promotes lipid accumulation during MASLD progression 6 (Figure 1). The authors first observed that YBX1 expression was elevated in liver samples from MASLD patients. Similarly, in the livers of high-fat-cholesterol and high-fructose diet-fed (HFCFD) mice, YBX1 expression was significantly upregulated and positively associated with increased lipid droplet accumulation, suggesting a potential correlation between YBX1 activity and MASLD. To test causality, the authors generated hepatocyte-specific YBX1-deficient (YBX1-KOhep) mice and found that YBX1 deficiency in hepatocytes leads to a substantial reduction of MASLD markers, characterized by a decrease in liver triglyceride content and lipid droplet size. These results confirmed that YBX1 contributes to lipid accumulation and liver steatosis. In an *in vitro* model of steatosis, induced by treating the immortalized mouse hepatocyte cell line AML12 with palmitic acid, YBX1 expression was consistently upregulated. Using this *in vitro* model, knockdown of YBX1 using shRNA targeting YBX1 (shYBX1) not only reduced lipid droplet formation but also impaired mitochondrial oxidative metabolism, as evidenced by decreased oxygen consumption rate (OCR), maximal respiration, and ATP production. These findings suggest that fatty acid exposure induces YBX1, which subsequently enhances both lipid uptake and metabolic processing in MASLD hepatocytes. Importantly, this study also reported the upregulation of CD36, a well-known fatty acid transporter, in both the *in vitro* model of steatosis and liver samples from MASLD patients and mice. Further ChIP-qPCR analysis using AML12 cells revealed that YBX1 promotes CD36 transcription by directly binding to its F1 promoter region. Moreover, CD36 overexpression via plasmid transfection in shYBX1-treated AML12 cells under steatotic conditions restored lipid droplet accumulation and reversed the reduction in maximal OCR caused by YBX1 knockdown.

Similarly, in YBX1-KOhep mice fed HFCFD, adeno-associated virus-mediated CD36 overexpression reversed the protective effects of YBX1 knockout on liver inflammation and lipid accumulation, as evidenced by increased lipid droplet formation in hepatocytes. Together, these *in vitro* and *in vivo* results suggest that CD36 is a key downstream mediator of YBX1's role in lipid accumulation. Surprisingly, CD36 overexpression also upregulated YBX1 expression and nucleus translocation via AKT activation in the shYBX1-treated AML12 cells under steatotic conditions, establishing a self-reinforcing positive feedback loop under MASLD conditions.

This is the first study to demonstrate that YBX1 promotes lipid accumulation in hepatocytes by transcriptionally enhancing CD36-mediated fatty acid uptake and metabolism in hepatocytes. Moreover, although not observed in basal conditions, a YBX1/CD36 positive feedback loop was observed in the *in vitro* model of steatosis, suggesting that lipid accumulation in MASLD may be a self-promoting process. However, the role of YBX1 in lipid metabolism and CD36 regulation appears to be cell-type dependent. For instance, as noted by the authors, contrary to the findings in hepatocytes presented here, a previous study demonstrated that YBX1 regulates CD36 expression post-transcriptionally and suppresses lipid uptake in macrophages 6, 7. Therefore, future studies are needed to demonstrate the role of YBX1 in MASLD pathogenesis in different cell types. Overall, the findings in this study deepen our understanding of MASLD pathogenesis and provide valuable insights for the development of therapeutic strategies targeting YBX1 or CD36.

## Figures and Tables

**Figure 1:**
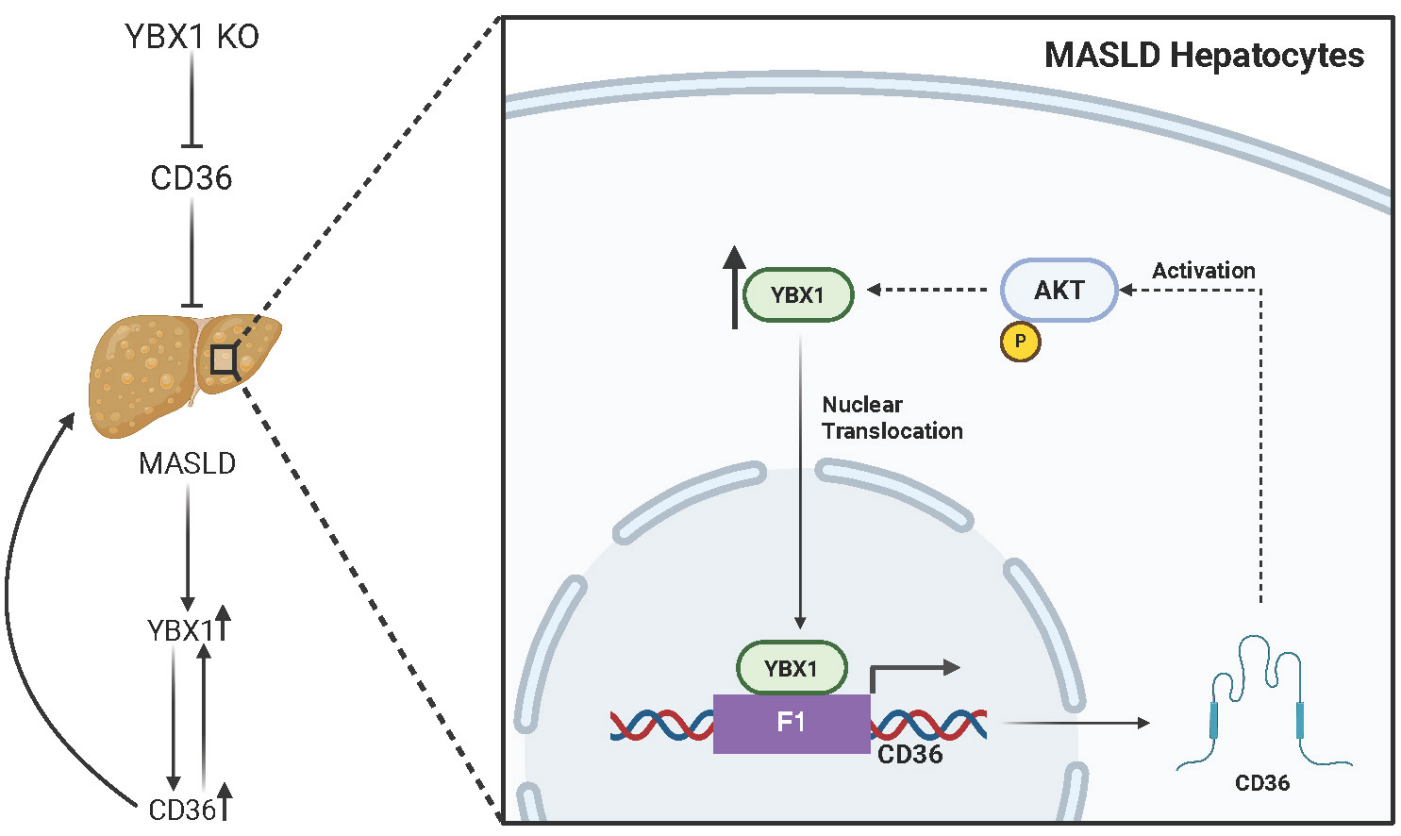
** YBX1/CD36 positive feedback loop in MASLD Hepatocytes.** Graphical illustration presenting the role of YBX1 in CD36-mediated lipid accumulation and MASLD development. Figure created with a BioRender license.
